# A Human Skin Model Recapitulates Systemic Sclerosis Dermal Fibrosis and Identifies *COL22A1* as a TGFβ Early Response Gene that Mediates Fibroblast to Myofibroblast Transition

**DOI:** 10.3390/genes10020075

**Published:** 2019-01-22

**Authors:** Tomoya Watanabe, DeAnna Baker Frost, Logan Mlakar, Jonathan Heywood, Willian A. da Silveira, Gary Hardiman, Carol Feghali-Bostwick

**Affiliations:** 1 Division of Rheumatology and Immunology, Department of Medicine, Medical University of South Carolina, 96 Jonathan Lucas St, MSC 637, Charleston, SC 29425, USA; nabetomo0968@yahoo.co.jp (T.W.); bakerde@musc.edu (D.B.F.); logan.mlakar@gmail.com (L.M.); heywood.jonathan@gmail.com (J.H.); 2 Center for Genomic Medicine, Bioinformatics, Medical University of South Carolina, Charleston, SC 29425, USA; willian.abraham@gmail.com (W.A.d.S.); G.Hardiman@qub.ac.uk (G.H.); 3 Departments of Medicine and Public Health Sciences, Medical University of South Carolina, Charleston, SC 29425, USA; 4 School of Biological Sciences & Institute for Global Food Security, Queens University Belfast, Belfast BT9 5AG, UK

**Keywords:** fibrosis, systemic sclerosis, *COL22A1*, fibroblasts, TGF

## Abstract

Systemic sclerosis (SSc) is a complex multi-system autoimmune disease characterized by immune dysregulation, vasculopathy, and organ fibrosis. Skin fibrosis causes high morbidity and impaired quality of life in affected individuals. Animal models do not fully recapitulate the human disease. Thus, there is a critical need to identify ex vivo models for the dermal fibrosis characteristic of SSc. We identified genes regulated by the pro-fibrotic factor TGFβ in human skin maintained in organ culture. The molecular signature of human skin overlapped with that which was identified in SSc patient biopsies, suggesting that this model recapitulates the dermal fibrosis characteristic of the human disease. We further characterized the regulation and functional impact of a previously unreported gene in the setting of dermal fibrosis, *COL22A1*, and show that silencing *COL22A1* significantly reduced TGFβ-induced *ACTA2* expression. *COL22A1* expression was significantly increased in dermal fibroblasts from patients with SSc. In summary, we identified the molecular fingerprint of TGFβ in human skin and demonstrated that *COL22A1* is associated with the pathogenesis of fibrosis in SSc as an early response gene that may have important implications for fibroblast activation. Further, this model will provide a critical tool with direct relevance to human disease to facilitate the assessment of potential therapies for fibrosis.

## 1. Introduction

Systemic sclerosis (SSc) is a multisystem connective tissue disease characterized by immune dysregulation, vasculopathy, and excessive fibrosis of the skin and internal organs due to fibroblast proliferation and production of extracellular matrix [1,2]. It has the highest disease-related mortality and morbidity among the rheumatologic illnesses with impaired quality of life. In fact, fibroproliferative illnesses are responsible for approximately 45% of deaths in developed countries [3]. The progression of organ fibrosis leads to end-stage organ failure as a result of the loss of normal structure and function. Skin fibrosis causes hardening and tightening of the skin. These features lead to limited joint movement or cause joint contractures, diminished mouth opening, and impaired quality of life. However, the mechanisms underlying skin fibrosis remain to be completely elucidated.

Collagen type XXII alpha 1 chain (*COL22A1*), which is present on human chromosome 8q24.2, exhibits a unique localization at the myotendinous junctions, tendons, heart, articular cartilage, and skin [4]. In the skin, *COL22A1* is expressed surrounding the lower third of the anagen hair follicles [4]. Furthermore, COL22A1 is known to act as a cell adhesion ligand for skin epithelial cells and fibroblasts [4]. It belongs to the FACIT (fibril-associated collagens with interrupted triple helix) subset of the collagen superfamily that includes type IX, XII, XIV, XIX, and XXI collagens [5]. These are quantitatively minor collagens that mediate ligand interactions between fibrils and their milieu. They associate with collagen fibers through their C-terminal collagenous domains, and they mediate protein-protein interactions through their N-terminal non-collagenous domains [5]. One of the functions of COL22A1 described in zebrafish is the stabilization of myotendinous junctions and the strengthening of skeletal muscle attachments during contractile activity [6]. Transcript levels of *COL22A1* are elevated in the head and neck squamous cell carcinoma (HNSCC) and are proposed as prognostic predictors for HNSCC [7]. In the setting of fibrosis, *COL22A1* was identified as a potential causal variant in patients with diffuse cutaneous SSc (dcSSc) through whole-exome sequencing (WES) [8]. *COL22A1* was significantly enriched in the extracellular matrix–related pathway. However, the regulation and potential role of COL22A1 in fibrosis and specifically in the pathogenesis of SSc remain unexplored. Since most studies involving the development of fibrosis have examined the effect of pro-fibrotic triggers in vitro in cells such as fibroblasts and in vivo in animal models, in this study, we sought to identify genes regulated by TGFβ in human skin and found *COL22A1* as a top regulated gene. We also investigated the role of COL22A1 in the activation of fibroblasts and the development of fibrosis.

## 2. Materials and Methods

Additional methods can be found in the Supplementary section.

### 2.1. Primary Human Skin and Lung Fibroblasts Culture

Primary fibroblasts were cultured from skin or lung tissues of healthy donors as previously described [9,10,11]. Lung fibroblasts were obtained under a protocol (#970946) approved by the Institutional Review Board (IRB) of the University of Pittsburgh, and skin tissues and fibroblasts obtained without identifiers were approved as non-human subject research by the IRB of the Medical University of South Carolina. Fibroblasts were maintained in Dulbecco’s Modified Eagle’s Medium (DMEM) (Mediatech, Herndon, VA, USA) supplemented with 10% fetal bovine serum (Sigma-Aldrich, St. Louis, MO, USA), penicillin, streptomycin, and antimycotic agent (Invitrogen, Carlsbad, CA, USA) and used in passages two to seven. Skin fibroblasts were treated with TGFβ (5 ng/mL) (R&D Systems, Minneapolis, MN, USA) and harvested 2, 4, 8, 16, 24, 48 h (for RNA), and 72 h (for protein) post-treatment. Lung fibroblasts were treated with TGFβ (10 ng/mL) and harvested 48 h (for RNA) and 72 h (for protein) post-treatment. A549 cells were treated similarly to lung fibroblasts.

### 2.2. Small Interfering RNA (siRNA) Transfection

Primary human skin fibroblasts were seeded at a density of 2 × 10^5^ cells per well in six well plates 24–48 h prior to transfection with siRNA. ON-TARGETplus *COL22A1*-specific siRNA and ON-TARGETplus control siRNA were purchased from Dharmacon (Lafayette, CO, USA). Transfection was done using Lipofectamine 2000 (Invitrogen) and 100 nmol siRNA diluted in Opti-MEM I Reduced-Serum Medium (Life Technologies, Carlsbad, CA, USA) following the manufacturer’s recommendation. TGFβ was added to media of cells 24 h after transfection. Fibroblasts were harvested 24 or 48 h (for RNA) and 72 h (for protein) post-treatment.

### 2.3. Ex Vivo Human Skin Culture

Normal human skin was obtained from residual tissue following plastic surgery. All tissues were obtained according to the guidelines of the Medical University of South Carolina Institutional Review Board without any identifiers. Subcutaneous fat tissue was removed. The skin was cut with a disposable biopsy 3 mm punch, and the pieces of tissue were cultured in medium containing TGFβ (10 ng/mL) or a vehicle control. The culture medium was DMEM supplemented with penicillin, streptomycin, and anti-mycotic agent (Invitrogen). Skin samples were cultured in an air-liquid interface with the epidermal side up. Skin tissues were harvested at 2, 4, 8, 16, 24, and 48 h (for RNA) or 72 h (for histological analysis) post-treatment.

### 2.4. Quantitative PCR

Total RNA was extracted from human skin tissues and cultured fibroblasts using the TRIZOL Lysis Reagent (Life Technologies) and RNeasy^®^ kit (Qiagen Inc., Valencia, CA, USA). Reverse transcription was performed with SuperScript IV (Invitrogen). Gene messenger RNA (mRNA) expression levels were evaluated using quantitative PCR by the TaqMan^®^ real-time PCR system (Life Technologies) according to the manufacturer’s protocol on a TaqMan^®^ Gene Expression Assays Step One Plus real-time PCR system (Life Technologies). Premixed PCR primers and TaqMan probes for human *COL1A1, COL22A1, CTGF, FN, ACTA2,* and *GAPDH* were obtained from Life Technologies. Gene expression levels were normalized to *GAPDH* and compared with the 2^−ΔΔ*C*t^ method.

### 2.5. Western Blot Analysis

Western blot analysis of fibroblast lysates and supernatants was done as previously described [11]. The following antibodies were used: Anti-COL22A1 (Novus, Littleton, CO, USA), fibronectin, collagen 1A1, CTGF, GAPDH (Santa Cruz, Dallas, TX, USA), ACTA2 (Sigma-Aldrich), and horseradish peroxidase-labeled secondary antibody (Santa Cruz). Signals were detected by chemiluminescence (ProteinSimple, San Jose, CA, USA).

### 2.6. RNA Expression Profiling

Total RNA was extracted from human skin tissues using TRIZOL Lysis Reagent (Life Technologies) and the RNeasy^®^ kit (Qiagen Inc.) and was processed by the MUSC Genomics core for 1 × 50 cycles, using single-end RNA sequencing on an Illumina HiSeq2500. The RNA integrity was verified on an Agilent 2200 TapeStation (AgilentTechnologies, Palo Alto, CA, USA). A total of 100 ng of total RNA was used to prepare RNA-Seq libraries using the TruSeq RNA Sample Prep kit following the protocol described by the manufacturer (Illumina, San Diego, CA, USA). Sequencing was performed on an Illumina HiSeq2500. Samples were demultiplexed using CASAVA (Illumina).

### 2.7. Bioinformatics and Statistical Analysis

For the differential gene expression analysis, we utilized the OnRamp’s advanced Genomics Analysis Engine as previously described [12]. We used human genome build GRCh37/hg19 as the reference and the following programs and versions: Fastqc version 0.11.5, cutadapt version 1.11, Star aligner version 2.5.2b, HTseq version 0.7.0, DESEq2 Version 1.18.1. A gene was considered differentially expressed (DE) if it was below the 0.4 adjusted p-value threshold cut-off as determined by the Benjamini-Hochberg false discovery rate (FDR) correction [13]. A systems-level analysis of the biological pathways affected by the DE genes was carried out using the iPathway Guide tool from Advaita^®^ Bioinformatics [14]. The dataset was deposited into the GEO repository under the accession number GSE109350.

### 2.8. Statistical Analysis

All continuous variables were expressed as the mean ± standard deviation. All statistical analyses were done using IBM SPSS statistics 22 (IBM Corporation, NY, USA). Statistical comparisons were performed using the unpaired Student’s *t*-test. Comparison of 3 or more groups was done using ANOVA with a post-hoc Tukey’s test to evaluate statistical significance.

## 3. Results

### 3.1. TGFβ Increases *COL22A1* Expression Ex Vivo and In Vitro

To identify new genes that may be involved in the development of dermal fibrosis mediated by TGFβ in human skin, we performed high-throughput RNA sequencing (RNA-seq) using ex vivo human skin samples treated with TGFβ or a vehicle control for 48 h. Gene expression profiling identified several novel transcripts in human skin tissues that were significantly upregulated by TGFβ including *COL22A1*, Prostate Transmembrane Protein, Androgen Induced 1 (*PMEPA1*), Dermatopontin (*DPT*), and others (Table 1). A complete list of all differentially-expressed genes regulated by TGFβ (530 using an FDR cutoff of 0.1, and 1051 when the FDR stringency was set at 0.4) is shown in Supplementary Table S1. A heatmap of the top 100 most significantly differentially expressed mRNAs as determined by DESeq2 (FDR < 0.1) is shown in Supplementary Figure S1. Pathway analysis suggested that the top impacted pathway was the ECM-receptor interaction, followed by Proteoglycans in cancer, Cell cycle, and Cytokine-cytokine receptor interaction (Supplementary Table S2).

**Figure 1:**
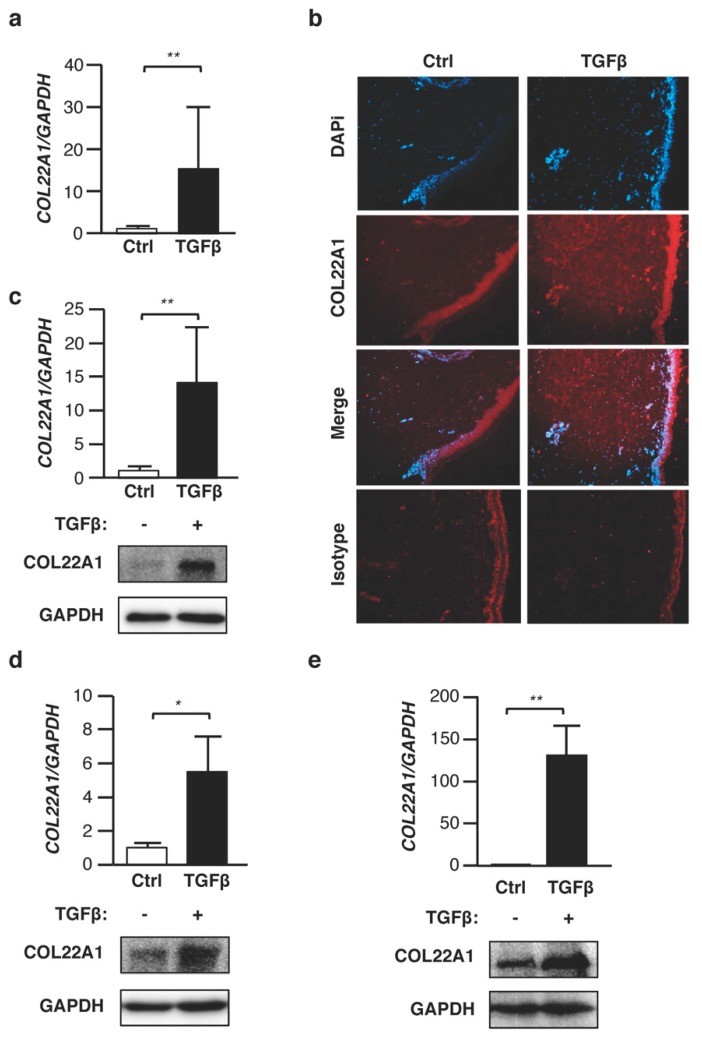
TGFβ increases expression levels of **COL22A1** ex vivo and in vitro. (**a**,**b**) Human skin samples were treated with TGFβ (10 ng/mL) for 48 and 72 h. (**a**) Expression levels of *COL22A1* were measured in human skin (N = 5); ** *p* < 0.01. (**b**) Localization of **COL22A1** in ex vivo normal skin. **COL22A1** was detected using immunofluorescence in a vehicle control or TGFβ-treated skin tissue. DAPI was used to detect nuclei (original magnification ×40); scale bars = 100 μm. (**c**) Human normal skin fibroblasts were treated with TGFβ (5 ng/mL) for 24 or 72 h. Expression levels of *COL22A1* mRNA were measured in human normal skin fibroblasts (N = 9); ** *p* < 0.01. Protein levels of **COL22A1** in the skin fibroblasts of three healthy donors were analyzed by immunoblotting of the lysates; GAPDH is shown as a loading control. (**d**) Human normal lung fibroblasts were treated with TGFβ (10 ng/mL) for 48 or 72 h. Expression levels of *COL22A1* mRNA were measured in human normal lung fibroblasts treated with a vehicle control or TGFβ (N = 3); * *p* < 0.05. The protein levels of COL22A1 in lung fibroblasts were analyzed by immunoblotting of the lysates. (**e**) A549 cells were treated with TGFβ (5 ng/mL) for 24 or 72 h. Expression levels of *COL22A1* mRNA were measured in A549 cells treated with a vehicle control or TGFβ (N = 3); ** *p* < 0.01. Protein levels of COL22A1 in the A549 cells were analyzed by immunoblotting of the lysates.

Among the differentially-regulated genes, *COL22A1* was the most highly regulated (fold change (FC) = 9.19, FDR = 6.40 × 10^−36^). Therefore, we focused on characterizing the levels and potential function of *COL22A1* in skin fibrosis. To confirm the results of the RNA-seq, we first examined the mRNA and protein levels of *COL22A1* in ex vivo human skin samples using quantitative reverse transcription PCR (qRT-PCR) and immunofluorescence, respectively. TGFβ significantly increased expression levels of *COL22A1* in human skin from different donors (Figure 1a). Immunofluorescence analysis also revealed COL22A1 protein in the dermal layer of human skin treated with TGFβ, whereas no COL22A1 was detected in human skin treated with the vehicle control (Figure 1b). Since fibroblasts are effector cells in dermal fibrosis [15], and having observed COL22A1 in the dermal layer of the skin, we next asked whether *COL22A1* expression is increased by TGFβ in skin fibroblasts. TGFβ significantly increased the expression of *COL22A1* in skin fibroblasts from healthy donors. This was confirmed at the protein level using Western blot analysis (Figure 1c). We also examined the ability of TGFβ to increase *COL22A1* expression in other cell types. Interestingly, mRNA and protein levels of COL22A1 were significantly increased by TGFβ in normal human lung fibroblasts (Figure 1d) and A549 cells (Figure 1e), which are adenocarcinomic human alveolar basal epithelial cells. Thus, TGFβ can induce *COL22A1* expression both in the skin- and lung-derived cells.

To further investigate the mechanisms involved in *COL22A1* expression, we examined whether other fibrosis-promoting growth factors, such as interleukin (IL-6), bleomycin (BLM), and Platelet-Derived Growth Factor (PDGF)-BB, induce *COL22A1* expression in dermal fibroblasts after 24 h. IL-6 and BLM showed modest increases in *COL22A1* expression in skin fibroblasts, while PDGF-BB had no effect (Supplementary Figure S2A,B).

### 3.2. COL22A1 Is a TGFβ Early Response Gene

**Figure 2:**
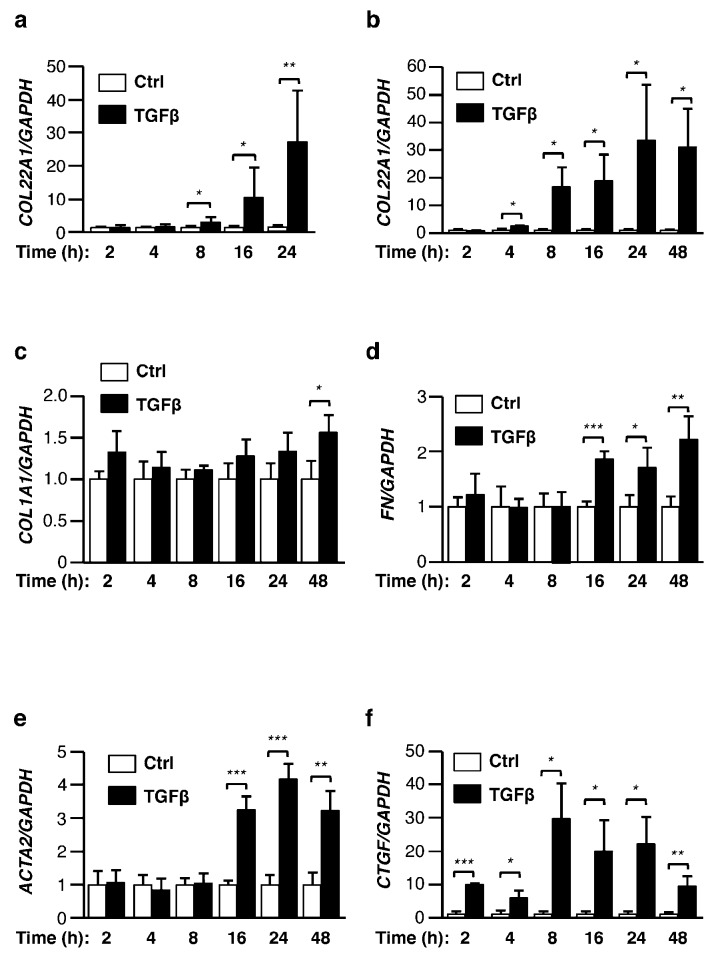
*COL22A1* is an early response gene ex vivo and in vitro. Human skin and fibroblasts treated with TGFβ (5 or 10 ng/mL) were harvested at the indicated time points. (**a**) The expression levels of *COL22A1* mRNA were measured in normal human skin in organ culture (N = 5); * *p* < 0.05; ** *p* < 0.01. (**b**) Expression levels of *COL22A1* mRNA were measured in primary human skin fibroblasts (N = 3): * *p* < 0.05. (**c**) *COL1A1* mRNA levels; * *p* < 0.05. (**d**) *FN* mRNA levels; * *p* < 0.05; ** *p* < 0.01; *** *p* < 0.001. (**e**) *ACTA2* mRNA levels: ** *p* < 0.01; *** *p* < 0.001. (**f**) *CTGF* mRNA levels: * *p* < 0.05; ** *p* < 0.01; *** *p* < 0.001.

To evaluate time-dependent changes induced by TGFβ, we measured the mRNA levels of *COL22A1* in ex vivo human skin samples. Human skin samples treated with TGFβ were harvested at 2, 4, 8, 16, and 24 h. TGFβ showed a time-dependent increase in *COL22A1* expression. mRNA levels of *COL22A1* were significantly upregulated starting at 8 h after stimulation with TGFβ (Figure 2a). We next compared time-dependent changes in normal skin fibroblasts. Similar to ex vivo human skin, significantly increased levels of *COL22A1* mRNA in skin fibroblasts were noted 4 h after TGFβ treatment (Figure 2b). This upregulation in skin fibroblasts was slightly earlier than that observed in ex vivo human skin. We further analyzed the expression of known fibrosis-related genes at the same time points. qRT-PCR analysis revealed that the expression levels of collagen type I alpha chain (*COL1A1)*, fibronectin (*FN*), and alpha 2 smooth muscle actin *(ACTA2)* were significantly increased at later time points (after 16 or 24 h of TGFβ treatment) compared with those of *COL22A1* (Figure 2c–e). In contrast, significant increases in connective tissue growth factor (*CTGF)* expression were observed as early as 2 h after TGFβ treatment (Figure 2f). Thus, TGFβ induces *COL22A1* expression earlier than other extracellular matrix genes and *ACTA2*, while the early response of the *COL22A1* expression is comparable to that seen with the early response gene *CTGF*.

### 3.3. TGFβ-Induced COL22A1 Expression Depends on ALK5 and MEK Activation

**Figure 3:**
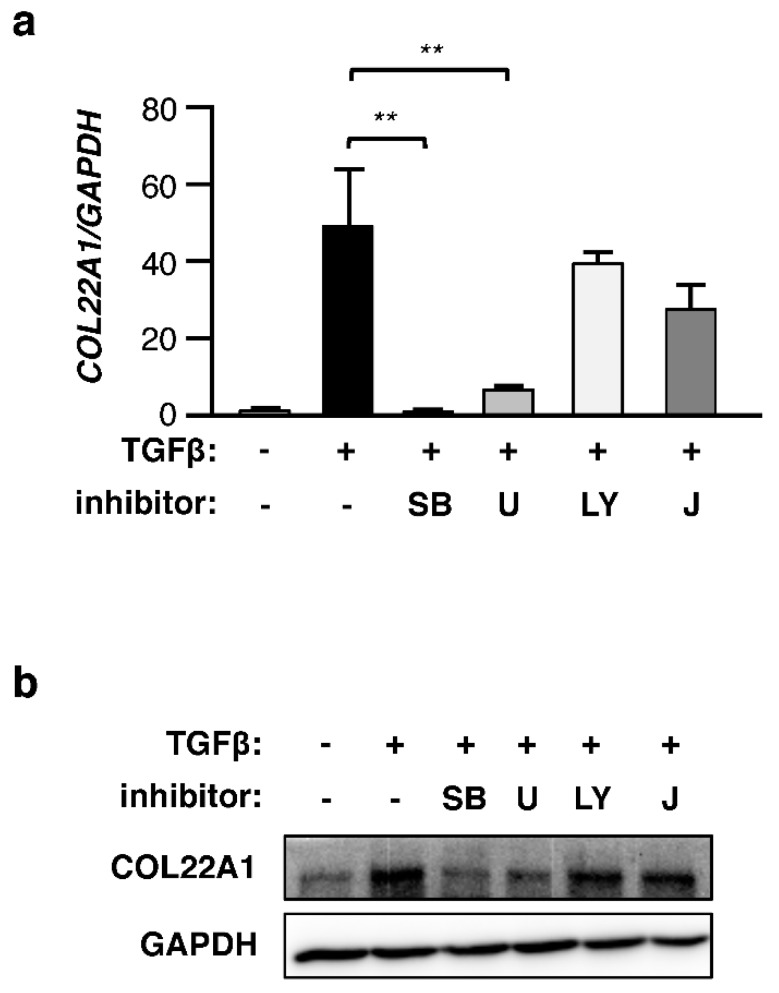
TGFβ-induced COL22A1 expression is inhibited by MEK and ALK5 inhibitors. Skin fibroblasts were incubated with 10 μmol/L SB431542 (SB), U0126 (U), LY294002 (LY), JNK inhibitor II (J), or DMSO as a vehicle control for 1 h before the addition of TGFβ (5 ng/mL). Skin fibroblasts were stimulated with TGFβ for 24 and 72 h. (**a**) Expression levels of *COL22A1* were measured using qPCR (N = 4); ** *p* < 0.01. (**b**) Representative data from immunoblotting of lysates are shown. GAPDH was used as a loading control.

To investigate which TGFβ signaling cascades are involved in the induction of *COL22A1* expression, normal skin fibroblasts were cultured with TGFβ in combination with specific inhibitors of ALK5 (SB431542), MEK (U0126), PI3K (LY294002), and JNK signaling. TGFβ induction of *COL22A1* expression was abrogated by ALK5 and MEK inhibition (Figure 3a). JNK inhibition partly blocked the TGFβ-induced expression of *COL22A1*. On the other hand, TGFβ-induced *COL22A1* expression was not affected by PI3K inhibition (Figure 3a). Similar effects were observed at the protein level (Figure 3b). These results suggest that the induction of *COL22A1* expression by TGFβ is mediated by ALK5 and MEK signaling.

### 3.4. Silencing COL22A1 Reduces TGFβ-Induced ACTA2 Expression

**Figure 4:**
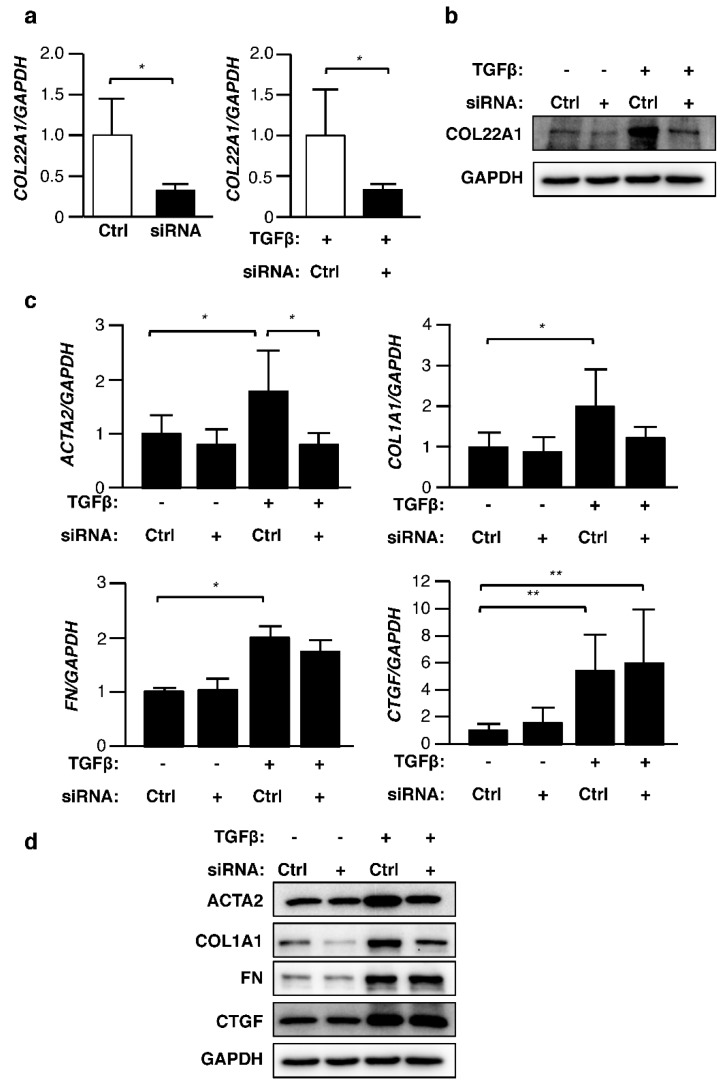
Silencing *COL22A1* reduces expression levels of ACTA2. Normal skin fibroblasts were transfected with control or *COL22A1* siRNA and treated with TGFβ (5 ng/mL) for 48 and 72 h (N = 4). (**a**) Expression levels of *COL22A1* mRNA were measured; * *p* < 0.05. (**b**) Protein levels of COL22A1 were analyzed by immunoblotting of lysates. GAPDH is shown as a loading control. (**c**) Expression levels of fibrosis-related genes were measured; * *p* < 0.05; ** *p* < 0.01. (**d**) Representative protein levels in lysates from fibroblasts transfected with control or *COL22A1* siRNA.

Since *COL22A1* expression was induced by TGFβ earlier than other genes, we examined the potential role of *COL22A1* knockdown on TGFβ regulation of other fibrosis-related genes. Normal skin fibroblasts were transfected with *COL22A1* siRNA and then stimulated with TGFβ. An average of 60% silencing of *COL22A1* was achieved in unstimulated fibroblasts compared to control siRNA (Figure 4a). *COL22A1* mRNA levels also decreased significantly in TGFβ-stimulated fibroblasts. Similarly, protein levels of COL22A1 were decreased by silencing *COL22A1* (Figure 4b). We further investigated whether other fibrosis related genes were affected by *COL22A1* knockdown. Interestingly, TGFβ-induced *ACTA2* expression was significantly decreased following *COL22A1* silencing (Figure 4c). This effect was also observed at the protein level (Figure 4d). *COL22A1* silencing reduced TGFβ induction of *COL1A1* and to a lesser degree that of *FN*, but the differences did not reach statistical significance. The protein levels of COL1A1 were also reduced. On the other hand, mRNA and protein levels of CTGF were not affected by *COL22A1* knockdown. These results indicate that COL22A1 may mediate TGFβ induction of ACTA2, and possibly COL1A1.

To determine whether *COL22A1* silencing alters the contractile capacity of dermal fibroblasts, we examined the effect of silencing *COL22A1* expression on dermal fibroblast-mediated collagen gel contraction. Our data show that *COL22A1* silencing in the absence of TGFβ modestly decreased the baseline gel contraction capacity of fibroblasts. However, *COL22A1* silencing did not significantly alter TGFβ-mediated collagen gel contraction of fibroblasts, even though it resulted in a pronounced reduction of TGFβ-induced *ACTA2* expression (Supplementary Figure S3).

### 3.5. *COL22A1* Expression Is Elevated in SSc Skin Fibroblasts

**Figure 5:**
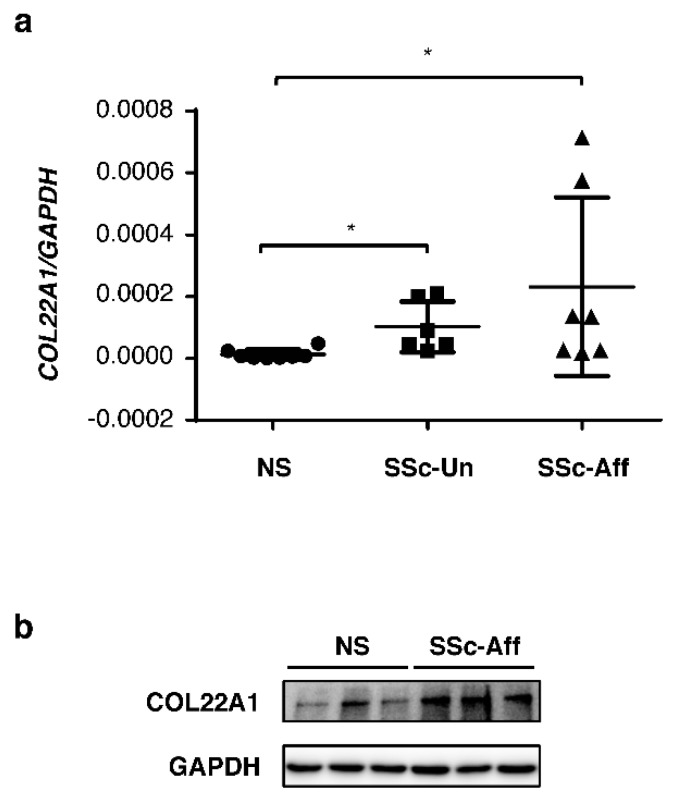
Increased expression levels of COL22A1 in SSc skin fibroblasts. Skin fibroblasts from healthy donors and patients with SSc were cultured in serum-free DMEM for 48 and 72 h. (**a**) Expression levels of *COL22A1* mRNA were measured in the indicated group (HC; N = 8, SSc-Un; N= 6, SSc-Aff; N = 7); * *p* < 0.05. (**b**) Protein levels of COL22A1 were detected in normal skin fibroblasts and SSc skin fibroblasts using immunoblotting. GAPDH is shown as a loading control. NS = normal skin fibroblasts; SSc-Un = SSc-unaffected skin fibroblasts; SSc-Aff = SSc-affected skin fibroblasts.

Since SSc is a disease characterized by dermal fibrosis, we compared mRNA and protein levels of COL22A1 in skin fibroblasts from control donors and patients with the diffuse cutaneous form of SSc. *COL22A1* expression was significantly increased in fibroblasts from lesional SSc skin (SSc-affected skin fibroblasts; SSc-Aff) and non-lesional SSc skin (SSc-unaffected skin fibroblasts; SSc-Un) compared to normal skin fibroblasts (Figure 5a). In contrast, no significant differences were found between affected and unaffected SSc fibroblasts. Protein levels of COL22A1 in SSc-affected fibroblasts were also higher than those in normal skin fibroblasts (Figure 5b). These findings suggest that *COL22A1* levels are elevated in fibroblasts from patients with SSc-associated dermal fibrosis, and due to its role in mediating TGFβ regulation of ACTA2 and myofibroblast differentiation, COL22A1 may be involved in the pathogenesis of SSc dermal fibrosis.

### 3.6. Genes Identified as Differentially Expressed in TGFβ-Treated Human Skin Overlap with SSc Patient Skin Signatures

**Figure 6:**
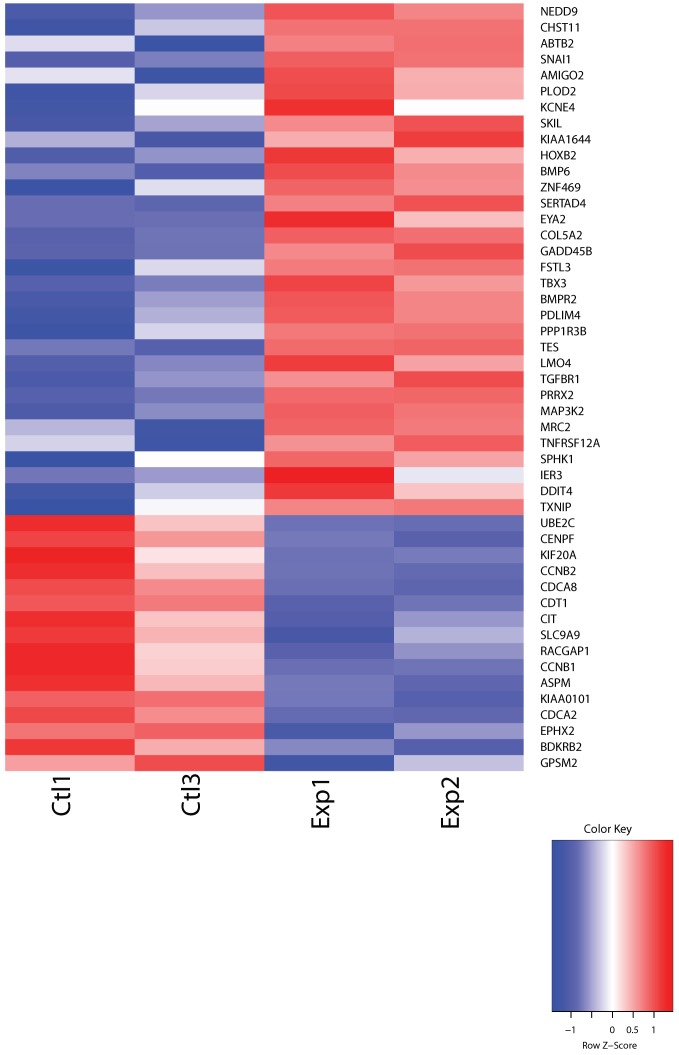
The heatmap of the 48-gene overlap between our study (significant DE mRNAs as determined by DESeq2 (FDR < 0.1)) and the 371 gene signature that differentiates the dSSc and the normal state described by Sargent et al. The red and blue boxes indicate relative over- and under-expression with respect to a reference which is calculated as the mid-point between the control and TGFβ treated groups.

To assess the similarity in the response of human skin in organ culture to a well-accepted signature of human SSc skin, we compared the gene signature identified in our model to that identified in primary fibroblasts stimulated with TGFβ as reported by Sargent et al. [16], and to the SSc skin signature identified by Milano et al. [17]. Sargent et al. carried out in vitro exposure of adult dermal fibroblasts from healthy and SSc patients and derived a gene signature for TGFβ responsiveness comprised of 674 unique genes. In their study, they reanalyzed a prior data set from Milano et al., derived from 75 arrays from patients with different disease severity. Although their 674 unique gene signatures distinguished the dSSc and healthy patients (53 arrays), this signature was noisy when applied to the extended data set with different disease progression represented. This signature was therefore refined to a smaller subset that better differentiated the dSSc and normal groups. We noted that these array 474 probes described by Sargent et al. corresponded to 371 genes. We looked for overlaps with all 1051 transcripts differentially expressed (FDR ≤ 0.4) in our study. To minimize technical noise associated with microarray experimentation and experimental differences between laboratories, we required that the direction of the fold change was the same between both studies, i.e., if a gene was reported as upregulated by Sargent et al., it also needed to be in this study for further consideration as a biomarker. This stringent filtering resulted in a 48 gene overlaps between our study and that of Sargent at al., and a heatmap of these transcripts which clearly distinguishes the control and TGFβ treated groups is presented in Figure 6. These findings suggest that our human skin ex vivo model is a suitable model for the dermal fibrosis characteristic of SSc.

## 4. Discussion

Research on fibrosis has relied on the use of in vitro assays and in vivo mouse and rat models. However, many of the agents tested in animals have not been efficacious in humans in clinical trials. Using human skin as an ex vivo organ model of fibrosis has great potential to examine mechanisms underlying fibrosis because this model more closely mimics the skin fibrosis characteristic of human SSc than the current in vitro and in vivo models. In fact, we have previously shown that factors such as IGFBP-5, IGFBP-3, and TGFβ can induce fibrosis in human skin maintained in organ culture [18,19,20]. We now show that our ex vivo human skin model is a suitable model for the dermal fibrosis characteristic of SSc. The signature obtained from the human skin stimulated with TGFβ and maintained in organ culture overlaps with the signature identified in two key studies using human dcSSc skin biopsies and human fibroblasts stimulated with TGFβ [16,17]. The 48-gene signature in the overlap between our model and these studies demonstrates the utility and relevance of our model for research focusing on dermal fibrosis in SSc.

RNA-seq using ex vivo human skin samples is a powerful tool for identifying new genes that may contribute to disease pathogenesis and their functional analysis. Using RNAseq, we identified the transcripts regulated by TGFβ in human skin, with *COL22A1* showing the greatest differential expression. Our findings reveal that in normal skin-derived fibroblasts, TGFβ significantly increased *COL22A1* expression. Furthermore, fibroblasts from the skin of dcSSc patients expressed high levels of *COL22A1* compared with normal skin fibroblasts. These findings suggest that upregulated *COL22A1* expression may be associated with the pathogenesis of fibrosis in patients with SSc and/or may be a biomarker for human dermal fibrosis. To the best of our knowledge, this study is the first to investigate the levels (both mRNA and protein) and the role of *COL22A1* in the setting of fibrosis.

Our analysis revealed that *COL22A1* is a TGFβ-inducible immediate early gene in skin fibroblasts while other fibrosis-promoting growth factors had a very modest effect on *COL22A1* expression. TGFβ has been shown to induce several downstream genes such as *CTGF*, *EGR-1*, and Endothelin-1 (*ET-1*) at an early stage compared with other fibrosis-associated genes [21,22,23]. These factors play an important role in the deposition of extracellular matrix components including fibronectin and collagen by fibroblasts in the development and maintenance of fibrosis. In fact, CTGF is an inducible matricellular protein involved in tissue remodeling, and its expression is increased during wound repair. CTGF mediates some of the effects of TGFβ, and CTGF transgenic mice were shown to have multi-organ fibrosis [24]. The fact that *COL22A1* expression is induced early while that of other ECM genes and *ACTA2* are increased in a delayed manner raises the possibility that COL22A1 may mediate the TGFβ induction of pro-fibrotic factors. Indeed, siRNA experiments showed that silencing *COL22A1* reduced *ACTA2*, *COL1A1*, and *FN* expression and late-response genes downstream of *TGFβ*, but not *CTGF* expression, which is induced earlier than *COL22A1*. These results lead us to speculate that *COL22A1* acts upstream of *ACTA2*, *COL1A1*, and *FN* and regulates their expression through the TGFβ signaling pathway.

In addition, *COL22A1* showed a higher magnitude of response to TGFβ than other TGFβ regulated genes we examined. Other fibrosis-associated genes such as *COL1A1, FN,* and *ACTA2,* which are late response targets of TGFβ, showed increased expression of 2–5-fold following TGFβ stimulation, whereas the early response genes such as *COL22A1*, *CTGF* (Figure 3f), and *EGR-1* [25] showed more than a 30-fold induction in expression in skin fibroblasts. This greater response may reflect the acute phase of the response to TGFβ, while the late response genes are more characteristic of the chronic response to TGFβ and other triggers of fibrosis. Although *COL22A1* silencing decreased TGFβ-mediated *ACTA2* expression and αSMA levels, it did not affect the contraction of fibroblasts in the collagen gel. αSMA is a component of stress fibers in fibroblasts; however, its inhibition is not sufficient to prevent contraction. In fact, αSMA knockout mice show a delay in skin wound healing, suggesting that αSMA contributes to wound healing but is not necessary [26]. Additionally, αSMA null mice retained the ability to form myofibroblasts and contractile forces in response to TGFβ [27]. This may be in part due to the fact that additional proteins can compensate for the absence of αSMA. For instance, in fibroblasts and myofibroblasts isolated from αSMA knockout mice, other actin forms were found at higher levels post-TGFβ simulation [27]. Additionally, non-muscle myosins colocalize with actin and participate in contraction during wound healing [28]. Thus, fibroblasts have compensatory proteins that may reduce the functional impact of the αSMA decrease on cell contraction. 

Regulation of *COL22A1* expression by fibrosis-promoting growth factors also appears to provide some insights into mechanisms regulating *COL22A1* expression by TGFβ. PDGF-BB did not increase *COL22A1* expression, while IL-6 and BLM modestly increased its levels. Our experiments using TGFβ signaling cascade inhibitors identified pathways regulating *COL22A1* expression. PDGF is known to result in the activation of JAK/STAT, PI3K, phospholipase C-γ (PLC-γ) or MAPK signaling pathways [29]. The binding of IL-6 to its receptor leads to the activation of JAK/STAT and Ras-mediated signaling [30]. In addition, IL-6 also activates PI3K [30,31]. Similar to these factors, BLM does not activate ALK5 or MEK signaling pathways. However, we showed that the activation of PI3K was not required to induce *COL22A1* expression at the mRNA and protein levels. In this regard, ALK5 and MEK signaling pathways seem to play a key role in mediating the TGFβ regulation of *COL22A1* expression, explaining, at least in part, why TGFβ was more potent for inducing *COL22A1* expression.

*COL22A1* is expressed in tissue junctions, tendons, heart, articular cartilage, and skin [4]. However, no studies to date have assessed *COL22A1* expression in lung tissue-derived cells. We demonstrate that the expression levels of *COL22A1* were increased by TGFβ in adenocarcinomic human alveolar basal epithelial cells and lung fibroblasts. These results suggest that that *COL22A1* may be associated with the development of lung fibrosis as well as skin fibrosis via increased expression in resident fibroblasts contributing to their activation and transition to myofibroblasts. Furthermore, TGFβ-induced *COL22A1* expression may induce epithelial mesenchymal transition (EMT) in human alveolar epithelial cells. EMT is a biologic process that involves an epithelial cell acquiring a mesenchymal cell phenotype, which includes enhanced migratory capacity, resistance to apoptosis, and increased production of ECM components [32]. In fact, TGFβ stimulation promotes alveolar epithelial cell transition to a myofibroblast-like phenotype in lung tissues [33]. EMT is also found to be associated with fibrosis occurring in different organs including the kidney, liver, lung, and intestine. Therefore, the high induction of *COL22A1* by TGFβ in A549 cells and lung fibroblasts, in conjunction with the reduction of *ACTA2* and *COL1A1* expression by *COL22A1* silencing, leads us to speculate that *COL22A1* may be associated with the transition of fibroblasts and epithelial cells to myofibroblasts, effector cells that play an important role in extracellular matrix production and fibrosis. In fact, in the skin, *COL22A1* is expressed surrounding the lower third of anagen hair follicles and acts as a cell adhesion ligand for skin epithelial cells and fibroblasts [4]. It has been reported that *ACTA2* is expressed in human hair follicles and that hair follicle-derived smooth muscle cells express *ACTA2* [34]. It is thus conceivable that hair follicle cells can be another source of the myofibroblasts characteristic of fibrotic skin. Furthermore, since hair follicles are involved in perifollicular fibrosis, and our findings suggest skin localization of COL22A1, it is likely that COL22A1 may also play an important role in perifollicular fibrosis and may promote cell adhesion and migration of hair follicle-derived smooth muscle cells.

In conclusion, our findings suggest that the increased expression of *COL22A1* is associated with the pathogenesis of fibrosis in SSc and that the regulation of *COL22A1* expression may have important implications for skin fibrosis as well as lung fibrosis. These results add to our understanding of the pathogenic mechanism of fibrosis by generating novel insights into genes regulated under pro-fibrotic conditions in human skin, providing direct relevance to human dermal fibrosis.

## Figures and Tables

**Table 1:** The significant differentially expressed (DE) messenger RNA (mRNAs) as determined by DESeq2 (false discovery rate, FDR < 0.1) using ex vivo human skin samples treated with TGFβ or a vehicle control for 48 h. The top 100 most significant DE mRNAs ranked by FDR (padj) are presented.

Symbol	GeneID	Description	Padj	Fold Change
*COL22A1*	169044	collagen, type XXII, alpha 1	6.40 × 10^−36^	9.19
*PMEPA1*	56937	prostate transmembrane protein, androgen induced 1	6.36 × 10^−33^	3.51
*DPT*	1805	dermatopontin	2.89 × 10^−29^	4.69
*LTBP2*	4053	latent transforming growth factor beta binding protein 2	6.31 × 10^−26^	3.18
*COL1A1*	1277	collagen, type I, alpha 1	2.49 × 10^−23^	3.11
*FN1*	2335	fibronectin 1	2.78 × 10^−20^	3.39
*MMP10*	4319	matrix metallopeptidase 10	6.47 × 10^−13^	2.76
*COL3A1*	1281	collagen, type III, alpha 1	5.68 × 10^−12^	2.34
*DHRS2*	10202	dehydrogenase/reductase (SDR family) member 2	6.16 × 10^−12^	4.55
*COL5A2*	1290	collagen, type V, alpha 2	8.53 × 10^−12^	2.36
*ITGB6*	3694	integrin, beta 6	1.66 × 10^−11^	2.94
*ADAMTS15*	170689	ADAM metallopeptidase with thrombospondin type 1 motif, 15	1.77 × 10^−11^	3.55
*LRRC15*	131578	leucine rich repeat containing 15	1.77 × 10^−11^	4.17
*TGFBI*	7045	transforming growth factor, beta-induced, 68kDa	6.21 × 10^−11^	3.12
*MKI67*	4288	marker of proliferation Ki-67	7.29 × 10^−11^	−2.56
*COL1A2*	1278	collagen, type I, alpha 2	8.85 × 10^−11^	2.11
*ADAM12*	8038	ADAM metallopeptidase domain 12	3.00 × 10^−10^	2.71
*GLS*	2744	glutaminase	3.08 × 10^−10^	2.08
*MIAT*	440823	myocardial infarction associated transcript (non-protein coding)	3.36 × 10^−10^	3.23
*C4orf26*	152816	chromosome 4 open reading frame 26	3.59 × 10^−10^	4.07
*WNT5B*	81029	wingless-type MMTV integration site family, member 5B	1.01 × 10^−9^	3.58
*KRT77*	374454	keratin 77, type II	3.56 × 10^−9^	2.19
*CXCL14*	9547	chemokine (C-X-C motif) ligand 14	7.49 × 10^−9^	2.61
*TPST1*	8460	tyrosylprotein sulfotransferase 1	8.75 × 10^−9^	2.58
*COL15A1*	1306	collagen, type XV, alpha 1	1.03 × 10^−8^	2.75
*SNAI1*	6615	snail family zinc finger 1	1.04 × 10^−8^	2.65
*NKD1*	85407	naked cuticle homolog 1 (Drosophila)	2.62 × 10^−8^	3.41
*LDLRAD4*	753	low density lipoprotein receptor class A domain containing 4	1.43 × 10^−7^	3.30
*NPR3*	4883	natriuretic peptide receptor 3	1.46 × 10^−7^	2.96
*DHRS9*	10170	dehydrogenase/reductase (SDR family) member 9	1.64 × 10^−7^	−2.44
*COL5A1*	1289	collagen, type V, alpha 1	2.11 × 10^−7^	2.11
*ESM1*	11082	endothelial cell-specific molecule 1	2.11 × 10^−7^	2.83
*DIRAS1*	148252	DIRAS family, GTP-binding RAS-like 1	2.17 × 10^−7^	2.51
*DLGAP5*	9787	discs, large (Drosophila) homolog-associated protein 5	3.77 × 10^−7^	−2.66
*RRM2*	6241	ribonucleotide reductase M2	7.85 × 10^−7^	−2.46
*TPM1*	7168	tropomyosin 1 (alpha)	7.85 × 10^−7^	2.55
*VCAN*	1462	versican	8.57 × 10^−7^	2.42
*CENPF*	1063	centromere protein F, 350/400kDa	9.28 × 10^−7^	−2.54
*CEP55*	55165	centrosomal protein 55kDa	1.84 × 10^−6^	−2.49
*SOX9*	6662	SRY (sex determining region Y)-box 9	1.84 × 10^−6^	1.93
*HECW2*	57520	HECT, C2 and WW domain containing E3 ubiquitin protein ligase 2	2.00 × 10^−6^	2.61
*PRSS23*	11098	protease, serine, 23	2.00 × 10^−6^	2.44
*SPRR2D*	6703	small proline-rich protein 2D	2.42 × 10^−6^	−1.78
*GADD45B*	4616	growth arrest and DNA-damage-inducible, beta	2.70 × 10^−6^	1.98
*KIF4A*	24137	kinesin family member 4A	2.84 × 10^−6^	−2.55
*CACNA1C*	775	calcium channel, voltage-dependent, L type, alpha 1C subunit	2.87 × 10^−6^	2.69
*LARP6*	55323	La ribonucleoprotein domain family, member 6	3.68 × 10^−6^	2.23
*MMP13*	4322	matrix metallopeptidase 13	3.83 × 10^−6^	3.02
*FGFBP2*	83888	fibroblast growth factor binding protein 2	5.18 × 10^−6^	2.93
*TOP2A*	7153	topoisomerase (DNA) II alpha 170kDa	6.21 × 10^−6^	−2.37
*DNAH17*	8632	dynein, axonemal, heavy chain 17	7.27 × 10^−6^	1.98
*IL11*	3589	interleukin 11	8.58 × 10^−6^	2.68
*EREG*	2069	epiregulin	9.06 × 10^−6^	−2.16
*PHLDB1*	23187	pleckstrin homology-like domain, family B, member 1	9.42 × 10^−6^	1.95
*IQGAP3*	128239	IQ motif containing GTPase activating protein 3	9.76 × 10^−6^	−2.42
*A2ML1*	144568	alpha-2-macroglobulin-like 1	9.83 × 10^−6^	−1.84
*NDC80*	10403	NDC80 kinetochore complex component	1.02 × 10^−5^	−2.57
*TROAP*	10024	trophinin associated protein	1.20 × 10^−5^	−2.45
*CDCA8*	55143	cell division cycle associated 8	1.40 × 10^−5^	−2.24
*TRIP13*	9319	thyroid hormone receptor interactor 13	1.89 × 10^−5^	−2.33
*GPRC5A*	9052	G protein-coupled receptor, class C, group 5, member A	2.19 × 10^−5^	2.37
*ARL15*	54622	ADP-ribosylation factor-like 15	2.51 × 10^−5^	2.32
*CDC20*	991	cell division cycle 20	2.68 × 10^−5^	−2.48
*BIRC5*	332	baculoviral IAP repeat containing 5	2.69 × 10^−5^	−2.34
*LYPD1*	116372	LY6/PLAUR domain containing 1	2.81 × 10^−5^	2.77
*DCLK3*	85443	doublecortin-like kinase 3	2.91 × 10^−5^	2.78
*AURKB*	9212	aurora kinase B	2.91 × 10^−5^	−2.28
*MYBL2*	4605	v-myb avian myeloblastosis viral oncogene homolog-like 2	3.49 × 10^−5^	−2.09
*MFAP2*	4237	microfibrillar-associated protein 2	3.53 × 10^−5^	1.88
*WNT2*	7472	wingless-type MMTV integration site family member 2	3.53 × 10^−5^	2.76
*S100A7A*	338324	S100 calcium binding protein A7A	3.77 × 10^−5^	−2.12
*NREP*	9315	neuronal regeneration related protein	3.86 × 10^−5^	1.92
*PKMYT1*	9088	protein kinase, membrane associated tyrosine/threonine 1	4.74 × 10^−5^	−2.07
*HMCN2*	256158	hemicentin 2	5.10 × 10^−5^	2.69
*CDC45*	8318	cell division cycle 45	5.23 × 10^−5^	−2.05
*TICRR*	90381	TOPBP1-interacting checkpoint and replication regulator	6.00 × 10^−5^	−2.40
*SETBP1*	26040	SET binding protein 1	6.31 × 10^−5^	1.87
*TPX2*	22974	TPX2, microtubule-associated	6.31 × 10^−5^	−2.25
*KIFC1*	3833	kinesin family member C1	6.49 × 10^−5^	−2.27
*CDCA5*	113130	cell division cycle associated 5	6.62 × 10^−5^	−2.12
*PRICKLE1*	144165	prickle homolog 1 (Drosophila)	7.37 × 10^−5^	2.23
*LBH*	81606	limb bud and heart development	8.71 × 10^−5^	2.31
*CCL28*	56477	chemokine (C-C motif) ligand 28	8.81 × 10^−5^	2.48
*FAM150B*	285016	family with sequence similarity 150, member B	8.81 × 10^−5^	2.65
*SKA3*	221150	spindle and kinetochore associated complex subunit 3	9.14 × 10^−5^	−2.41
*PRC1*	9055	protein regulator of cytokinesis 1	9.24 × 10^−5^	−2.12
*TACC3*	10460	transforming, acidic coiled-coil containing protein 3	9.34 × 10^−5^	−1.96
*KIF18B*	146909	kinesin family member 18B	1.05 × 10^−4^	−2.25
*CD83*	9308	CD83 molecule	1.09 × 10^−4^	2.07
*TMEM86A*	144110	transmembrane protein 86A	1.09 × 10^−4^	−1.88
*RUNX1*	861	runt-related transcription factor 1	1.18 × 10^−4^	1.99
*ATP12A*	479	ATPase, H+/K+ transporting, nongastric, alpha polypeptide	1.25 × 10^−4^	−2.26
*MAP1B*	4131	microtubule-associated protein 1B	1.25 × 10^−4^	1.71
*BGN*	633	biglycan	1.30 × 10^−4^	2.43
*PNMAL1*	55228	paraneoplastic Ma antigen family-like 1	1.31 × 10^−4^	2.25
*TK1*	7083	thymidine kinase 1, soluble	1.42 × 10^−4^	−2.08
*BMP6*	654	bone morphogenetic protein 6	1.46 × 10^−4^	2.19
*HPSE*	10855	heparanase	1.61 × 10^−4^	−1.77
*SEMA7A*	8482	semaphorin 7A, GPI membrane anchor (John Milton Hagen blood group)	1.73 × 10^−4^	1.78
*CCL8*	6355	chemokine (C-C motif) ligand 8	1.82 × 10^−4^	−2.56

## Supplementary Materials

The following are available online at http://www.mdpi.com/2073-4425/10/2/75/s1, Figure S1: Heatmap of significantly differentially expressed mRNAs as determined by DESeq2 (FDR < 0.1) using ex vivo human skin samples treated with TGFβ (10 ng/mL) or a vehicle control for 48 h, Figure S2: Human normal skin fibroblasts were treated with IL-6 (20 ng/mL), PDGF-BB (40 ng/mL), or BLM (10 mU/mL) for 24 or 72 h, Figure S3: Decreased expression of *ACTA2* in TGFβ-treated fibroblasts deficient in *COL22A1* does not alter TGFβ-induced collagen gel contraction, Table S1: Significantly DE mRNAs as determined by DESeq2 using ex vivo human skin samples treated with TGFβ (10 ng/mL) or a vehicle control for 48 h, Table S2: The most significantly impacted biological pathways due to TGFβ treatment, ranked by adjusted p value from most significant to least significant, Supplemental method 1: Immunofluorescence, Supplemental method 2: Fibroblast treatment, Supplemental method 3: Collagen Gel Contraction Assay.
