# Supplementary material for: A Human Skin Model Recapitulates Systemic Sclerosis Dermal Fibrosis and Identifies COL22A1 as a TGFβ Early Response Gene that Mediates Fibroblast to Myofibroblast Transition
Source: Genes (Basel). 2019 Jan 22;10(2):75. doi: 10.3390/genes10020075 (PMC6409682; doi:10.3390/genes10020075)
Supplement: Supplementary file 1 [file genes-10-00075-s001.zip › genes-414882-SI.pdf]

Watanabe et al Supplemental information

Supplemental Figures

Figure S1: Heatmap of significantly differentially expressed mRNAs as determined by DESeq2 (FDR <0.1) using *ex vivo* human skin samples treated with TGFβ (10 ng/ml) or a vehicle control for 48 hours. The top 100 most significant DE mRNAs ranked by FDR are presented. Red and blue boxes indicate relative over- and under-expression with respect to a reference which is calculated as the mid-point between the control and TGFβ treated groups.

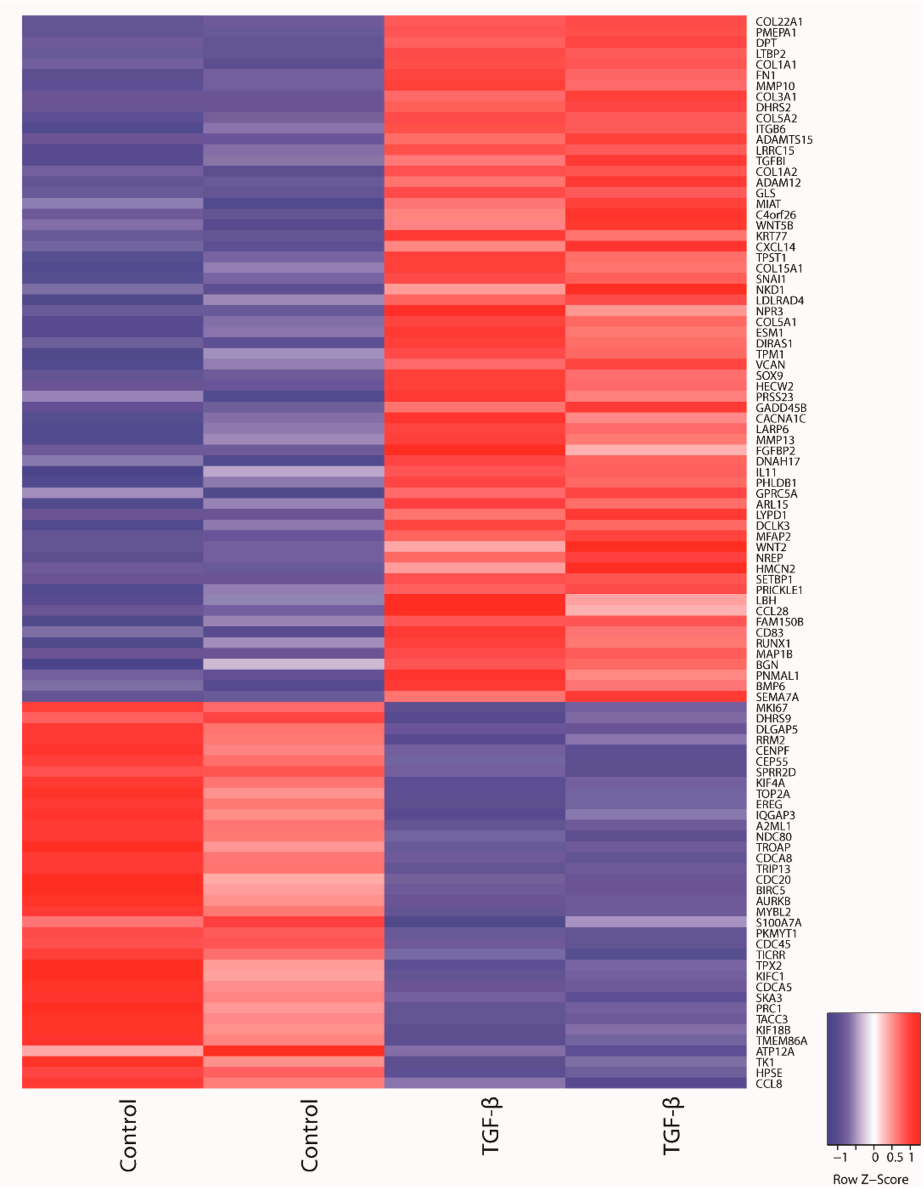

Figure S2. Human normal skin fibroblasts were treated with IL-6 (20 ng/ml), PDGF-BB (40 ng/ml), or BLM (10 mU/ml) for 24 or 72 hours. (a) Expression levels of *COL22A1* mRNA following stimulation with the indicated fibrosis growth factors were measured in normal skin fibroblasts (N = 3). (b) Protein levels of COL22A1 in fibroblasts stimulated with the indicated fibrosis growth factors were analyzed by immunoblotting of lysates.

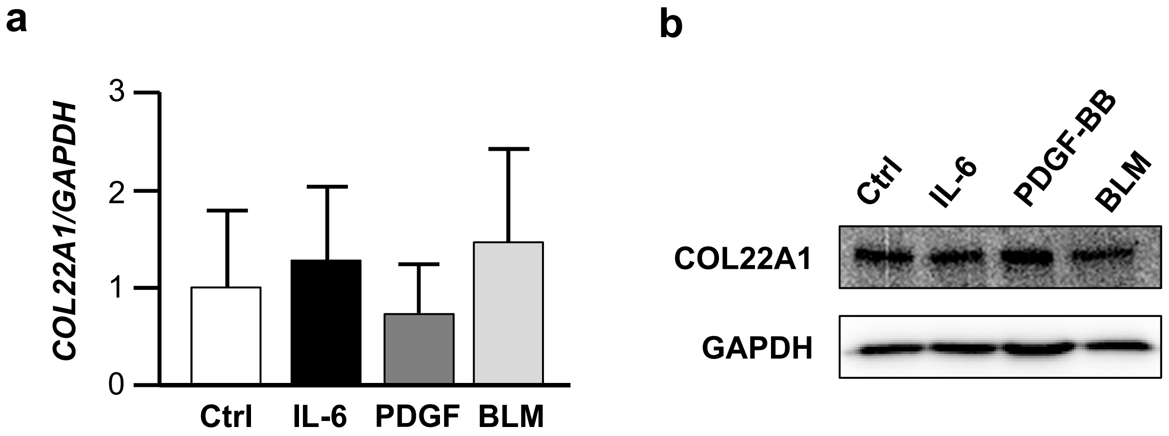

Figure S3: Decreased expression of *ACTA2* in TGFβ-treated fibroblasts deficient in *COL22A1* does not alter TGFβ-induced collagen gel contraction. Human normal foreskin fibroblasts were transfected with control siRNA (Ctrl) or *COL22A1(+)* siRNA for 48 hr and used in a gel contraction assay with or without TGFβ for 72 hr. (a) *ACTA2* mRNA levels. (b) Collagen gel

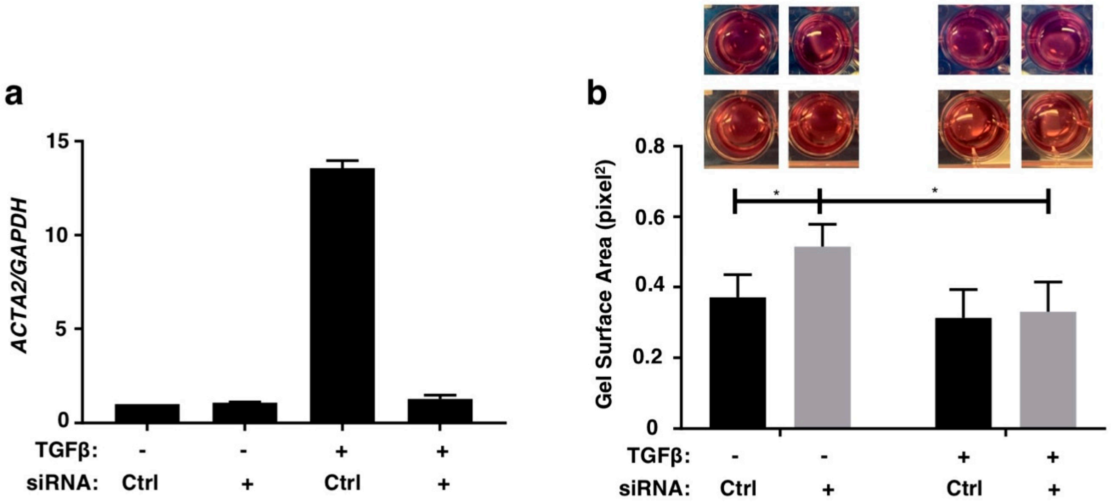

surface area. Representative images of wells from adult dermal and foreskin fibroblasts are shown as examples. * $p < 0.05$.

Supplemental Tables

Table S1: Significantly DE mRNAs as determined by DESeq2 using *ex vivo* human skin samples treated with TGFβ (10 ng/ml) or a vehicle control for 48 hours. Transcripts were sorted according to their padj-value, which is the smallest false discovery rate (FDR) at which the gene is called significant. 530 mRNAs were significant at an FDR of 0.1. When the stringency was released to 0.4, 1,051 transcripts were significant. The baseMean is a mean value for the counts from the various control and treated samples. The log2FoldChange and FoldChange represent the logarithmic base 2 and linear fold change differences between control and TGFβ treated fibroblasts. The lfcSE is the standard error estimate for the log2 fold change estimate.

(provided as an excel file)

Table S2: The most significantly impacted biological pathways due to TGFβ treatment, ranked by adjusted p value from most significant to least significant.

pName	pv_fdr
ECM-receptor interaction	5.99276×10^{-06}
Proteoglycans in cancer	1.70104×10^{-05}
Cell cycle	1.70104×10^{-05}
Cytokine-cytokine receptor interaction	6.43141×10^{-05}
HTLV-I infection	2.48×10^{-04}
Focal adhesion	3.62×10^{-04}
Hippo signaling pathway	3.74×10^{-04}
Basal cell carcinoma	4.65×10^{-04}
Pathways in cancer	6.24×10^{-04}
Protein digestion and absorption	1.16×10^{-03}
Arachidonic acid metabolism	1.66×10^{-03}
PI3K-Akt signaling pathway	1.66×10^{-03}
Amoebiasis	1.66×10^{-03}
Hypertrophic cardiomyopathy (HCM)	1.80×10^{-03}
Sphingolipid metabolism	1.97×10^{-03}
Phagosome	3.25×10^{-03}
Breast cancer	3.85×10^{-03}
Small cell lung cancer	3.85×10^{-03}
Antigen processing and presentation	3.85×10^{-03}
Axon guidance	4.16×10^{-03}
DNA replication	6.69×10^{-03}
Melanogenesis	6.69×10^{-03}

Complement and coagulation cascades	7.56×10^{-03}
Rheumatoid arthritis	1.33×10^{-02}
Dilated cardiomyopathy	1.33×10^{-02}
mTOR signaling pathway	1.49×10^{-02}
Wnt signaling pathway	1.49×10^{-02}
Cell adhesion molecules (CAMs)	1.59×10^{-02}
Type I diabetes mellitus	2.38×10^{-02}
Allograft rejection	2.72×10^{-02}
p53 signaling pathway	2.74×10^{-02}
Glycosphingolipid biosynthesis - lacto and neolacto series	2.82×10^{-02}
Staphylococcus aureus infection	3.33×10^{-02}
Arrhythmogenic right ventricular cardiomyopathy (ARVC)	3.33×10^{-02}
Graft-versus-host disease	3.43×10^{-02}
Oocyte meiosis	4.48×10^{-02}
Regulation of actin cytoskeleton	5.52×10^{-02}
Systemic lupus erythematosus	5.71×10^{-02}
Signaling pathways regulating pluripotency of stem cells	5.71×10^{-02}
Steroid biosynthesis	5.84×10^{-02}
Viral myocarditis	5.87×10^{-02}
VEGF signaling pathway	6.08×10^{-02}
Synthesis and degradation of ketone bodies	6.21×10^{-02}
Autoimmune thyroid disease	7.18×10^{-02}
Ovarian steroidogenesis	7.68×10^{-02}
Terpenoid backbone biosynthesis	7.92×10^{-02}
Chagas disease (American trypanosomiasis)	8.03×10^{-02}
Herpes simplex infection	8.98×10^{-02}
AGE-RAGE signaling pathway in diabetic complications	9.73×10^{-02}

Supplemental Methods:

Immunofluorescence. Skin was fixed with 10% formalin and embedded in paraffin. Six micrometer sections of paraffin-embedded skin specimens were deparaffinized in xylenes and rehydrated in graded solutions of ethanol. Antigens were retrieved using citric acid based antigen retrieval solution (Vector Lab. Inc., Burlingame, CA). Immersing the sections in BLOXALL Blocking Solution (Vector Lab. Inc., Burlingame, CA) quenched endogenous peroxidases for 30 minutes. The sections were permeabilized using 1% donkey serum in 1x PBST for 20 minutes. Nonspecific interactions were blocked using 5% donkey serum in 1x PBST. Sections were blocked with 1% serum and incubated with polyclonal anti-COL22A1 antibody (Abcam, Cambridge, MA) or IgG isotype control (Invitrogen) overnight at 4°C. Sections were washed with 1% serum in 1X PBST and incubated with Alexa Fluor 555–conjugated donkey anti-rabbit IgG (Invitrogen, CA, USA) for 1 hour. ProLong™ Diamond Antifade Mountant with DAPI

(Thermo Fisher Scientific, Lafayette, CO) was used to identify nuclei. Images were captured on an Axio Observer Microscope (Carl Zeiss Microscopy GmbH, Germany) using identical settings.

Fibroblast treatment. Kinase inhibitors LY294002, U0126 (Cell Signaling Technology, Danvers,MA), SB431542, and JNK inhibitor II (Sigma-Aldrich, St. Louis, MO) were added to media of cells 1 hour before TGFβ stimulation. Skin fibroblasts were treated with recombinant human IL-6 (20 ng/ml) (R&D Systems, Minneapolis, MN), platelet-derived growth factor BB-isoform (PDGF-BB, 40 ng/ml) (R&D Systems, Minneapolis, MN), and bleomycin (BLM, 10 mU/ml) (Nippon Kayaku, Tokyo, Japan) and harvested 24 hours (for RNA) and 72 hours (for protein) post-treatment.

Collagen Gel Contraction Assay. Fibrobasts were plated at $1.5x10^5$ in a 6-well culture dish. After transfection with siRNA, the cells were detached using Accutase Cell Detachment Solution (BD Biosciences, San Jose, CA) and washed with 1X PBS then resuspended in serum-free, 1X DMEM at $1.5x10^5$ cells/mL. Three parts of the cell suspension was mixed with two parts of the collagen solution at 3mg/mL, diluted in 0.1% acetic acid (Corning, Corning, NY) with NaOH. The cell-collagen solution was plated in a 24-well culture dish and incubated at room temperature for 20 minutes to allow it to solidify. TGFβ or vehicle was diluted in 1X, serum-free DMEM and carefully layered on top of the cell-collagen matrix. The matrix was immediately released using a 200 µL pipet tip. Images of the gel were taken over time and the area of cell-collagen matrix was measured using ImageJ.
